# Current Understanding of How Extracorporeal Membrane Oxygenators Activate Haemostasis and Other Blood Components

**DOI:** 10.3389/fmed.2018.00352

**Published:** 2018-12-12

**Authors:** Andrew J. Doyle, Beverley J. Hunt

**Affiliations:** Thrombosis and Haemophilia Centre, Guy's and St Thomas' NHS Foundation Trust, London, United Kingdom

**Keywords:** Haemostasis, ECMO, cardiopulmonary bypass, ventricular assist device, thrombosis, hemorrhage

## Abstract

Extracorporeal membrane oxygenators are used in critical care for the management of severe respiratory and cardiac failure. Activation of the coagulation system is initiated by the exposure of blood to synthetic surfaces and the shear stresses of the circuit, especially from device pumps. Initial fibrinogen deposition and subsequent activation of coagulation factors and complement allow platelets and leucocytes to adhere to oxygenator surfaces and enhance thrombin generation. These changes and others contribute to higher rates of thrombosis seen in these patients. In addition, bleeding rates are also high. Primary haemostasis is impaired by platelet dysfunction and loss of their key adhesive molecules and shear stress causes an acquired von Willebrand defect. In addition, there is also altered fibrinolysis and lastly, administration of systemic anticoagulation is required to maintain circuit patency. Further research is required to fulyl establish the complexities of the haemostatic changes with these devices, and to elucidate the mechanistic changes that are mainly responsible so that plans can be made to reduce their complications and improve management.

## Background

Non-biological artificial materials, or biomaterials, are used increasingly to carry blood in medical devices, and much work has gone in to improving morbidity and mortality associated with their use. Extracorporeal circulation is used to manage patients with severe organ dysfunction. Several devices are routinely used with extracorporeal surfaces—extracorporeal membrane oxygenation (ECMO), haemodialysis, left ventricular assist devices (LVAD), and cardiopulmonary bypass (CPB). In all these devices blood comes into constant contact with blood leading to activation of the coagulation system, platelets, leucocytes and complement, which is then returned to the patient. This subsequently may lead to thrombosis, bleeding and device failure, which remain frequent despite increasing knowledge of their pathogenesis. Further understanding of their haemostatic changes will allow the development of improved function and lifespans of the devices and reduce the development of systemic complications for patients.

ECMO is currently used to provide gas exchange in severe respiratory failure with veno-venous (VV) circuits and increasingly with concurrent cardiac failure via a veno-arterial (VA) circuit whilst waiting for organ recovery to occur. The circuit consists of a minimum of two large bore central cannulae which are connected to a membrane oxygenator device, which has a large biomaterial surface area coming into contact with blood, and a pump. The pump can be centrifugal or pulsatile in nature and rates vary due to the type of circuit used causing damage to cells and proteins passing through it. CPB has a similar circuit design to ECMO although with additional reservoirs used for giving therapeutic cardioplegia and return of autologous blood. VAD is a pump with an external extracardiac connection between the ventricle and aorta or pulmonary artery to bypass those with end-stage heart failure. Similar to ECMO and CPB, they have high shear stress but significantly less surface area and have different modes of pulsatility through the pump.

**Figure 1:**
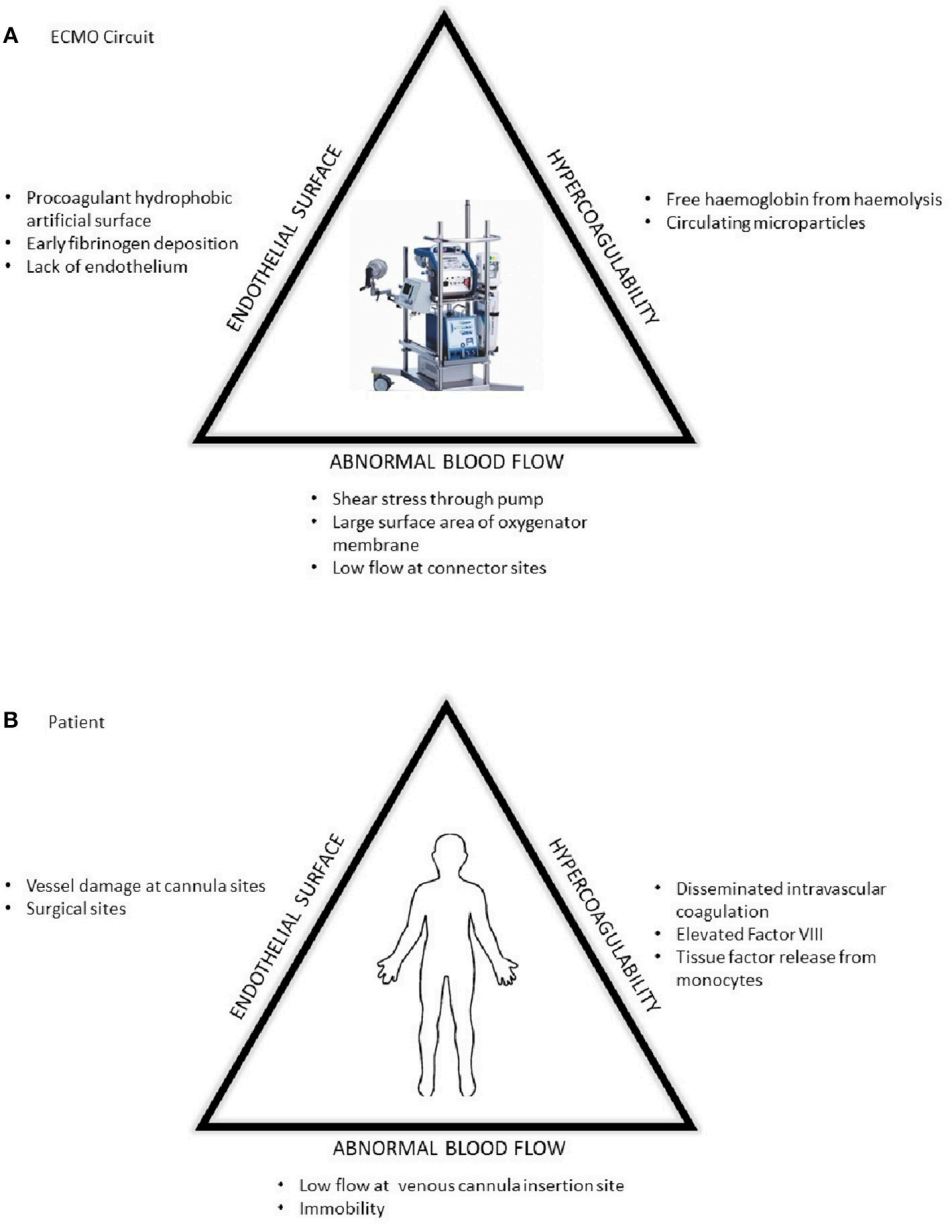
The prothrombotic changes described by Virchow's triad in respect to **(A)** the ECMO circuit and **(B)** patient factors.

At present, ECMO is associated with significant haemostatic challenges with clinical thrombosis and hemorrhage rates higher than the general critical care population. There is an added complication of circuit loss due to intra-device thrombosis particularly around the oxygenator membrane (Table 1). These problems are superimposed on other haemostatic changes in the patient due to their underlying disease state and treatment which may include the haemostatic effects of renal and hepatic failure, and a procoagulant acute phase response in those with infective and inflammatory illnesses and disseminated intravascular coagulation (DIC). The presence of DIC in patients preceding ECMO has a signficantly increased risk of death (1) (Figure 1). Rates of reported ECMO-associated venous thromboembolism (VTE) range from 18 to 85% in different centers and may be at least partly dependent on anticoagulation regimens (2, 3). Oxygenator thrombosis can occur in around 10–16% of patients depending on the circuit type and age of the patient (4, 5). Severe hemorrhage is reported in nearly 40% and intracranial hemorrhage in 16–21% of patients (6–8). Haemostatic complications are associated with poor patient survival outcomes (4).

**Table 1:** Clinical manifestations caused by the activation of coagulation and blood components with ECMO and causative changes.

	**Clinical manifestations**	**Potential causative changes**
Thrombosis	Deep vein thrombosis Pulmonary embolism Oxygenator thrombosis Small vessel thrombosis	Increased coagulation factors Contact pathway activation Haemolysis and free hemoglobin Vessel injury at cannulae sites Microthrombi formation Circulating microparticles Pre-existing systemic inflammation in patients e.g. Monocytic tissue factor
Hemorrhage	Line and surgical site Pulmonary and upper airway Intracranial Abdominal	von Willebrand Factor dysfunction Increased fibrinolysis Thrombocytopenia Platelet dysfunction and damage Reduced coagulation factors Hypofibrinogenaemia Systemic anticoagulation
Inflammatory response	Systemic inflammatory response syndrome Capillary leak syndrome	Complement activation Neutrophil and monocyte activation Contact pathway activation

Extracorporeal surfaces can consist of a variety of manufactured materials including polyethylene, polypropylene and silicone which are designed to withstand the pressures and demands of the associated devices. However, they not haemostatically inert leading to subsequent haemostatic disturbances. There are two approaches to managing this—reducing the surfaces ability to activate haemostasis and using systemic inhibitors of coagulation such as anticoagulation and antiplatelet agents. Today, most ECMO and CPB circuits are heparin-coated which have been shown to reduce platelet, leucocytes and coagulation activation (9).

Modern anticoagulation is principally achieved using an unfractionated heparin infusion for ECMO and CPB although other intravenous agents such as bivalirudin and argatroban are increasingly popular. They must can be considered in those with heparin-induced thrombocytopenia, heparin resistance or allergy but are also used outside of these indications (10, 11). At present, there is wide variation in practice, and no consensus on the administration and monitoring of anticoagulation during ECMO and the management of ECMO-related hemorrhage and VTE (12). The aim of current anticoagulation is to reduce thrombin generation however it also increases the risk of hemorrhage. The ideal therapeutic agent would reduce the thrombotic risk without increasing the rate of bleeding, but this is not possible with current anticoagulants.

ECMO and CPB have similarities in their main function and have a similar exposure to biosurfaces and shear stress enabling extrapolation of data between the two devices. However, there are several differences between them that one must be aware of prior to inferring results. These include the duration of ECMO lasting for day to weeks as opposed to minutes and hours for CPB. Also, the level of anticoagulation is higher during CPB than ECMO with addition of protamine reversal in the former. Furthermore, patients undergoing CPB have therapeutic hypothermia and haemodilution with a large number of surgical sites. The clinical phenotype of the patients undergoing ECMO are those with acute critical illness usually with a generalized inflammatory response and associated coagulopathy whereas most patients have to be fit for anesthesia for CPB but have chronic severe cardiac disease. Patients with VAD have end-stage cardiac failure and are used as a bridge to transplant or to allow for myocardial recovery. They have significantly reduced surface area in comparison to CPB and ECMO for coagulation activation, but the use of a pump still causes shear stress to molecules and cells that pass through them.

We aim to explore the present data suggesting how the haemostatic system becomes activated with ECMO from the current research that is available in comparison to CPB and VAD. The implications that these have upon the risk of thrombosis and hemorrhage in these patients and the impact on their management will be discussed.

## Initial Interactions Between Coagulation Factor and Extracorporeal Biosurfaces

**Figure 2:**
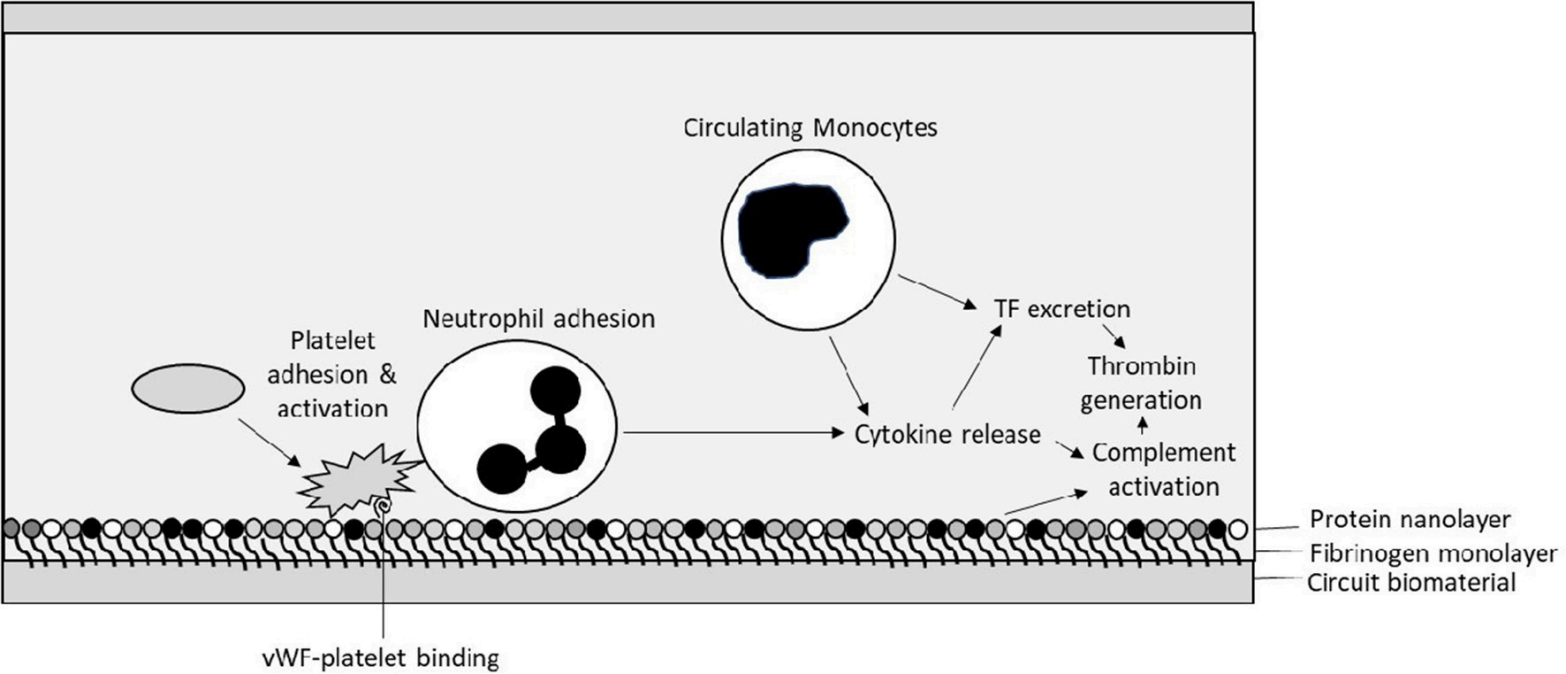
Initial interactions between circuit biosurface, haemostatic factors, and components of blood—Tissue Factor (TF), von Willebrand factor (vWF).

The interaction between biomaterial surfaces and blood has been extensively described by Vroman et al. who reviewed the interaction principally between glass surfaces and protein adsorption over 30 years ago (13). The subsequent “Vroman effect” has been described as the sequential adsorption of proteins to a biomaterial surface. Initial immediate adsorption of fibrinogen occurs to the surfaces within minutes of exposure (13). Next, a variety of proteins then bind to the fibrinogen nanosurface (Figure 2). These include the coagulation factors of the contact activation pathway—high molecular weight kininogen (HMWK) and factor XII–as well as high-density lipoprotein (HDL), albumin, immunoglobin G (IgG), and complement component C3 (14). It has been shown by Passmore et al. that fibrinogen levels fall in an ovine lung injury model with the commencement of the ECMO circuit and recover to the baseline level within 48 h in keeping with its adhesion to the biosurface and loss from circulation (15). We have also observed this acquired hypofibrinogenaemia in pediatrics following CPB with increased hemorrhage and reduced clot strength (16).

After the formation of a protein-based layer on the surface the ECMO circuit cellular adhesion occurs. Platelets and polymorphonuclear leucocytes (PMN) interact with each other on the protein-covered biomaterial surface. Interaction with the complement system also contributes to their activation. This results in propagating further adhesion and PMN release of cytokines including interleukins-1β, -6, and -8 and tumor necrosis factor-α, potentially contributing further to a pro-inflammatory state (17).

## Properties of Biomaterials and Their Interaction With Blood Components

Different components of the ECMO circuit are designed to have varying properties depending on their use such as tubing, oxygenator membranes, and connectors. The oxygenator membranes are of importance as they have a large surface in contact with blood. They are made of materials such as polymethyl pentene and polypropylene, which are hydrophobic. This allows gas exchange to occur but stops blood from passing through the membrane pores. This results in a prothrombotic tendency due to the adsorption of the unwanted proteins, known as protein fouling, which then undergo further conformational changes that also make them more thrombogenic. The nature of protein adsorption depends on the biomaterial surface and alters further protein-cellular interactions and cellular adhesion. For example, HDL adsorption is higher to hydrophobic surfaces with reduced platelet adhesion but surfaces with higher fibrinogen adsorption have more platelet adhesion (14, 18).

To reduce protein fouling and the prothrombotic nature of the ECMO surfaces, small molecules can be incorporated in to the biosurface coating. Initially, molecules such as albumin and silicone have been used to act as a barrier to coagulation factors and complement but still cause a similar degree of thrombocytopenia (19). Further development led to heparin-coating, which is widely used in commercially-available ECMO circuits, providing an additional localized anticoagulant effect. Heparin-coating decreases thrombin generation by binding to circulating antithrombin and demonstrates less platelet and leucocyte activation (20–22). The development of this approach has reduced thrombosis and filter losses although they still occur (23). Currently available ECMO circuits with different coatings do not show any significant difference in haemostatic parameters indicating there is little evidence of heparin leaching off the surfaces (24).

Current developments to further reduce protein fouling include the coating of high density hydrophilic molecules, which bind high amounts of water, and to include polymers with branch chains to stop conformational changes to the adsorbed proteins, known as polymer brushes (25, 26). Obstals et al. have described the development of an polymer brush coated surface for ECMO, which significantly decreased coagulation, platelet, and leucocyte activation *ex vivo* (27). At present, these are not used clinically due to difficulties in the polymerisation process.

## Activation of the Coagulation Cascade in Extracorporeal Circulation

The cell-based model of coagulation suggests that it is initiated by the exposure of subendothelial tissue factor (TF) binding and activating circulating FVII which then through the coagulation cascade causes downstream thrombin generation. In disseminated intravascular coagulation due to sepsis further TF expression on monocytes and macrophages occurs and needs to be considered in patients receiving ECMO as sepsis is a common accompanying problem. The cell-based model does not include the intrinsic “contact activation” pathway coagulation factors (FXI, HMWK, prekalikrein, and FXII) which *in vitro* react with negatively charged artificial surfaces to cause thrombus formation. There is increasing interest in these coagulation factors and their role in the activation of coagulation by artificial surfaces.

There is a lack of data regarding the extent of TF-FVII as compared to contact activation mediated thrombin generation in ECMO. However, it has been demonstrated in CPB circuits and with synthetic surfaces that there is an increased in monocyte-related TF expression over several hours with an associated increase in procoagulability *in vitro* (28, 29). This is probably due to yet another mechanism—the adherence and subsequent activation of PMNs of the biomaterial surface with an associated fall in these circulating cells over this period. TF-mediated monocytic procoagulability has additionally been shown to be reduced by the heparin-coating of biomaterials which is now routinely used in most ECMO circuits (30).

Although the contact pathway coagulation factors do not play a principal role in haemostasis as a result of vessel injury, conflicting data indicates that thrombosis with biomaterials is related to Factor XII-mediated activation of coagulation (31, 32). In patients undergoing CPB, there is evidence of reduced circulating FXII, increased FXIIa and prekalikrein levels in association with increasing circulating FXII complexes although this was not replicated by others (33, 34). Plötz et al. showed in 10 neonates receiving ECMO that there was activation of the contact pathway with elevated FXII-C1 esterase inhibitor complexes, decreased kallikrein inhibitory capacity, and increased thrombin-antithrombin complexes (35).

Larsson et al. have described the use of a monoclonal anti-factor XIIa antibody, 3F7, that binds to the active site of FXII reducing its enzymatic activity. 3F7 has been used in an animal model with an ECMO circuit showing a significant reduction in arterial and venous thrombus formation without increasing the rate of bleeding (36). Similarly, May et al. have demonstrated that the use of a FXII inhibitor, rHA-infestin-4, reduced the rate of thrombosis formation similar to that of heparin in animal models using AV-shunts but as expected, it did not impact upon bleeding times that were similar to those without anticoagulation (37). This seems an attractive option for the anticoagulation of ECMO circuits however this has not yet been reproduced in humans in a similar setting (32).

There is minimal data studying other coagulation factors during ECMO. Factor VIII is an acute phase molecule that is significantly elevated in pro-inflammatory states. Passmore et al. showed that FVIII falls in both a lung-injury model and control-animal models on ECMO circuits than those without after 24 h by over 50% (15). This was concurrently noted to be with lower fibrinogen and von Willebrand factor (VWF) levels suggestive of increased consumption of FVIII or decreased VWF-binding ability. This fall of FVIII has not been seen in CPB although decreased level of factor X and prothrombin by 40–50% over 24 h have been shown with a proportional fall in thrombin generation (38). Kreyer et al. showed an association with lower FVII and FX levels in patients receiving ECMO who developed severe bleeding than those who did not (7).

There can be variations in analytical interpretation in the coagulation assays in these groups depending upon the biosurfaces, the anticoagulation regimen used, and the methods used for assessing coagulation. Certainly, increased activated circulating coagulation factors, increased fibrin degradation product, and detectable levels of circulating factor-inhibitor complexes are found but as there is no coherent explanation for the results, more research is needed.

## Activation of the Complement System in Extracorporeal Circulation

The complement system is activated by three initiating pathways dependent on the recognition of non-self-antigens: the classical (CP), lectin (LP), and alternative (AP) pathways. The CP and LP are initiated by antibody-antigen complexes and certain carbohydrates, respectively, which can be present in patients receiving ECMO who may have an underlying infectious process. The AP can be activated by the biomaterial surface directly with also a degree of self-activation via the amplification loop. Both immunoglobulin and C3b can bind to the fibrinogen monolayer formed on the surface of the biomaterial leading to activation of the complement activation pathway. These then ultimately trigger the common pathway with the activation of C3 leading to the formation of the membrane attack complex (MAC). As artificial surfaces, unlike the endothelium have no regulatory molecules for supressing the complement system, they lead to the propagation and positive feedback of the complement cascade leading to an excessive inflammatory response and capillary leak syndrome which has been demonstrated as a complication of extracorporeal circuits (17, 39).

Vallhonrat et al. showed a rapid increase in the complement degradation products, C4d, Bb, iC3b and SC5-9b, 1 h after commencing ECMO in two adult patients (40). There was a predominant increase in Bb formation suggestive of complement activation by the AP. These markers subsequently fell within the next few hours and normalized 2 days later in keeping with a significant complement activation from initial exposure to the ECMO circuit. Similar results have been replicated *in vivo* with neonates and *in vitro*, although with a prolonged elevated of SC5-9b levels, and in patients on CPB and haemodialysis (41–44). Heparin has an anticomplement effect, so unsurprisingly heparin-bound surfaces in ECMO circuits have shown complement activation is reduced with maintenance of the complement regulatory proteins, Factor H & C1 Inhibitor (39).

The complement degradation products, anaphylatoxins, can either enter the liquid phase causing systemic inflammatory problems for patients or adhere to the ECMO surfaces leading to localized cellular activation principally of the innate immune system. The deposition of anaphylatoxins on the biomaterial surface can then have various implications on haemostasis. iC3b, a degradation product of C3b, can bind to a biosurfaces, and activate a specific granulocyte receptor, CR3, causing adhesion of principally neutrophils and to lesser extent, of monocytes. These then subsequently become activated (45). In keeping with this, it has been demonstrated that there is extensive PMN deposition over the surface of ECMO circuits that had been used in patients with evidence of cellular activation (46).

Complement cleavage products and anaphylatoxins that enter the systemic circulation have multiple interactions with various components of the coagulation system in particular FXI and FXII. They have been shown to activate platelets, increase TF expression, activate of endothelial cells increasing VWF release and enhancie the exposure of P-selectin on both platelets and activated endothelial cells (47, 48). It has also been shown that leucocyte activation occurs within hours of exposure to an ECMO circuit demonstrated by elevation of CD18, L-selectin, and neutrophil-derived elastase which can subsequently result in capillary leakage and pulmonary oedema (29, 49, 50).

## Activation of Platelets and Von Willebrand Factor in Extracorporal Circulation

As suggested previously, platelets adhere to the protein-coated monolayer of the biosurface of the ECMO circuit and react with other activated components of the coagulation and complement system systemically. It has been shown that thrombocytopenia is common in patients receiving ECMO although this is not related to its duration (1, 51). Severe thrombocytopenia is associated with multi-organ failure and preceding thrombocytopenia. 22% of patients receiving ECMO can have a severe thrombocytopenia of < 50 × 10^9^/L making adequate anticoagulation difficult and increasing the use of transfusion requirements (51).

Exposure to elevated shear flow from the ECMO circuit has been demonstrated to cause platelet receptor shedding of the key platelet adhesion glycoproteins, GPIbα, and GPVI and an associated loss of high molecular weight VWF multimers (HMWM) (52). The binding of the platelet GP1b-GVI complex and VWF is the main process of platelet adhesion to a fibrin-covered surface. This persists despite platelet transfusions and throughout the period of ECMO use. Subsequently, there are lower levels of platelet aggregation shown by light aggregometry using various agonists including ADP, ristocetin, collagen and epinephrine (52–56). This may contribute to the increased levels of bleeding seen in these patient in addition to the use of anticoagulation and antiplatelet agents. Flow cytometry of platelets in those receiving ECMO showed severely reduced expression of membrane-bound p-selectin and CD63, which modulates platelet spreading (56).

Despite having reduced aggregation and expression of the key platelet adhesion and structural molecules, Cheung et al. showed that there is a time-dependent platelet activation as measured by increased circulating matrix metalloproteinase (MMP)-2 and soluble p-selectin levels. This was not associated with significant activation of the endothelium (53). It suggests that platelet adhesion and aggregation is defective in those receiving ECMO but platelet activation occurs with the release of their granules.

In addition to platelet receptor shedding, the ECMO circuit can potentially induce the formation of thrombi and platelet microparticles. Although this has not been shown in ECMO, Danwanjee et al. have demonstrated in animal models that CPB can contribute to the formation of microthrombi in the liquid phase and deposit in various organ shown at biopsy with indium radio-labeling. No significant macroscopic thrombi nor significant occlusive material within the bypass apparatus were shown (57). These microthrombi are likely to form around the biosurface then subsequently embolise systemically. It has been shown the microthrombi may cause subsequent neurological sequelae and memory impairment in those who have received CPB (58).

Circulating platelet microparticles (PMP) have been demonstrated to be increased in extracorporeal circulation (59). PMP are small cell-derived particles typically 0.1–1 micrometres in size that are produced from activated platelets in situations of shear stress (60). They can act as a prothrombotic surface and due to their size have been shown to be increased systemically at the time of extracorporeal circulation (57). It is not clear whether PMP are important in the pathogenesis of ECMO coagulopathy and at present there is no information if they are correlated to a prothrombotic phenotype.

vWF activity decreases with time on ECMO (61–63). This is due to the loss of high molecular weight vWF multimers (HMWM) due to the effect of shear stresses in the circuit disrupting vWF molecules in the circuit and resolves with cessation. This is also seen with VAD (56, 64). Acquired von Willebrand's syndrome can be demonstrated serologically by a significantly reduced ristocetin cofactor activity and collagen-binding ability as well as a loss of HMWM. This has been correlated to an increased in bleeding typically from the respiratory tract, mucosal surfaces and puncture sites (56). Vincent et al. have shown that devices with a high shear have increased levels of proteolysis without the presence of endothelium in an *in vitro* circuit. However, in the presence of devices with high pulsality, this stimulates the release of VWF from the recipient's endothelium (65). This is may be a reason why higher bleeding rates with centrifugal pumps are seen in comparison to roller pumps in patients requiring ECMO (26.1 vs. 9.0 events/1000 treatment days, respectively) (66). Kalbhenn et al. have demonstrated after the cessation of ECMO that VWF parameters largely normalize after a few hours and completely within 24 h (55).

## Activation of the Fibrinolytic System in Extracorporeal Circulation

Fibrinolysis is essential for haemostatic regulation with the degradation of fibrin complexes once vessel injury has resolved. Hyperfibrinolysis is an overwhelming activation of this system leading to a bleeding phenotype which can be seen in trauma-induced coagulopathy, during liver transplantation and in some cancer patients. The release of tissue plasminogen activator (t-PA) can be stimulated by a number of factors including hypoxia, thrombin, histamine, and vasopressin. t-PA subsequently activates plasminogen to plasmin causing fibrin (and fibrinogen) breakdown.

The role of the fibrinolytic system in ECMO is not well described. McVeen et al. showed that there an initial significant fall in tPA and PAI-1 levels with a subsequent rise in both after several hours of ECMO then normalization over the next few days (67). These elevated fibrinolytic enzymes were also replicated in babies on ECMO >30 days of age with significant bleeding (68). Increases of tPA and D-dimer occur in patients undergoing CPB, with reduced bleeding, and clinical outcomes improved by using antifibrinolytic agents (69, 70).

## Effect of Haemolysis on Haemostasis in Extracorporeal Circulation

Red blood cell (RBC) breakdown (haemolysis) is routinely seen with ECMO circuits with 67% of patients showing increased levels of free circulating haemoglobulin (71). This occurs with higher flow rates of typically >147 ml/kg/min. The occurrence of significant haemolysis in ECMO is associated with a higher use of blood products. Free plasma hemoglobin levels of >50 mg/dL after 24 h have been shown to an independent predictor for mortality in ECMO (72).

Haemolysis causes prothrombotic changes as has been shown in other haemolytic conditions including sickle cell disease and autoimmune haemolytic anemia which are also associated with the presence of free hemoglobin and heme. Free hemoglobin is known to bind to nitric oxide further upsetting the haemostatic mileau by removing NO and therefore causing vasoconstriction and increasing platelet reactivity (73). Meyer et al. showed that free hemoglobin increased platelet adhesion to fibrinogen and collagen-coated biosurfaces with vWF. They felt that this was a result of increased binding strength between the vWF A1-domain and the platelet GP1bα receptor (74). Although there is a reduced level of multimeric vWF with ECMO as discussed previously, there are maintained levels of other multimer sizes that may contribute to platelet adhesion to biosurfaces.

In addition, RBC-derived microparticles can be formed as a result of ECMO-mediated haemolysis or from transfused blood products. RBC microparticles can increase thrombin generation as a result of phosphatidylserine exposure (75).

## Conclusions

It is apparent that the coagulopathy associated with ECMO is complex and is not fully elucidated. As we have discussed, it is clear that there are multiple interactions with varying defects in different haemostatic mechanisms, which ultimately lead to an increased risk of thrombosis and hemorrhage. Despite developments in the biomaterials with varying molecules embedded into the surface, there is still significant activation of haemostasis and the shear stresses of circuit pump will continue to contribute to the damage of blood-borne cells and large proteins.

Current literature suggests that an acquired von Willebrand syndrome, platelet dysfunction and activation of fibrinolysis are likely to be contributing to the increased hemorrhage rates seen in ECMO in addition to therapeutic anticoagulation. In parallel activation of both the extrinsic and contact-pathway coagulation factors, complement and circulating free hemoglobin promote thrombosis and thus circuit failure and systemic thromboembolism. It is still unclear at what time points in the use of the ECMO circuit that these changes have most clinical effect, and which contribute most significantly to its pathophysiology.

At present, much of what is known about ECMO is extrapolated from other devices in particular CPB but is not fully translatable. Further investigation is need on the haemostatic changes in ECMO to reflect what is known about other extracorporeal circuits. This needs to be applied to patient outcomes to identify patients at risk of thrombosis or bleeding, to guide targeted monitoring and management and develop alternative anticoagulation therapies. With these insights, it will then be possible to improve survival and decrease the morbidity associated with extracorporeal circuits.

## Author Contributions

AD performed the relevant literature search and wrote the review article. BH reviewed the article for submission and provided additional points for discussion.

### Conflict of Interest Statement

The authors declare that the research was conducted in the absence of any commercial or financial relationships that could be construed as a potential conflict of interest.
